# Silicosis mortality in Spain (1999–2020): A temporal and geographical approach

**DOI:** 10.3934/publichealth.2024036

**Published:** 2024-06-12

**Authors:** Germán Sánchez-Díaz, Greta Arias-Merino, Elisa Gallego, Rodrigo Sarmiento-Suárez, Verónica Alonso-Ferreira

**Affiliations:** 1 Institute of Rare Diseases Research, Instituto de Salud Carlos III, Av. Monforte de Lemos 3–5, Pabellón 11, Planta 1, 28029 Madrid, Spain; 2 Centro de Investigación Biomédica en Red de Enfermedades Raras, Av. Monforte de Lemos 3–5, Pabellón 11, Planta 0, 28029 Madrid, Spain

**Keywords:** mortality, silicosis, spatial epidemiology, time trends, occupational exposure, geographic analysis, mortality cluster

## Abstract

**Background:**

Silicosis is an occupational respiratory disease linked to silica dust inhalation. The main driver was traditional coal mining, but in recent decades, new sources of exposure have emerged. Our aim in this study was to assess the temporal and spatial distribution of mortality due to this disease over a 22-year period in Spain.

**Methods:**

Silicosis records, as an Underlying Cause of Death, were extracted from the National Institute of Statistics from 1999 to 2020 using the International Classification of Diseases 10th revision (code J62.8). Age- and sex-adjusted mortality rates per 1,000,000 inhabitants were calculated for the territory and by province. A geographic analysis was performed, and clusters of deaths were identified at the municipal level, and then the outcomes were compared in two periods of 11 years.

**Results:**

There were 2618 deaths due to silicosis in Spain. The mean age of death increased significantly by 0.66% annually from 1999 to 2013. The age-adjusted mortality rate decreased by 7.30% per year, falling from 3.00 to 0.65 per 1,000,000 inhabitants. The temporal pattern showed a significant decrease of mortality rate in 31% of the provinces (16 out of 52), while it increased in Pontevedra. Regarding the spatial analysis, 11 clusters were found in both periods, but some variations were observed in terms of their distribution in the Spanish territory, as well as in the affected municipalities.

**Conclusions:**

The decrease in mortality due to Silicosis could be related to less exposure to silica dust over the years and an improvement in the survival of those affected. It is thus essential to analyze the role of preventive measures for this occupational disease.

## 1. Introduction

Silicosis is a lung disease caused by the accumulation of silica dust crystals inside the respiratory cavity. This pneumoconiosis is primarily caused by occupational exposures and cannot be reversed once the disease is contracted [1]. Abundant in the earth's crust, silica is used in numerous industrial activities that involve cutting, crushing, and pulverizing this material through various methods. Therefore, workers are at risk of inhaling silica particles if preventive measures are not taken, even though this may not cause an immediate health effect [2]. The number of workers exposed to silica dust is very significant worldwide, estimated at 1.7 million in the USA, between 3 and 5 million in the European Union, and 3 million in India [3].

There are different clinical forms of silicosis depending on the duration of exposure to silica dust [4]. The most frequent is simple silicosis, which usually appears after 10 years of exposure. In contrast, accelerated and acute silicosis can occur after shorter periods due to high levels of silica dust, and it may be related to tobacco use or tuberculosis [1],[5],[6].

More than 23,000 incident cases of silicosis were reported worldwide in 2017, showing significant variations between continents [7]. Globally, the prevalence of silicosis has decreased annually by 4.6% between 1990 and 2019, being this decline more pronounced in high-income countries (24.9%) than in low-middle and low-income countries (3.5% and 5.7% respectively) [8].

Regarding mortality, the number of deaths from silicosis indicates its current status as a global health problem. An estimated 12,886 deaths were reported in 2019, making it the most prevalent pneumoconiosis, surpassing asbestosis (3,571) or coal workers' pneumoconiosis (3007) [9]. However, it is noteworthy that mortality rates have been decreasing since 1990, especially in the European region. The worldwide crude mortality rate has declined from 0.28 to 0.17 per 100,000 between 1990 and 2019, with a decrease of 41% [9]. Notably, substantial differences exist between countries based on income. In low-income regions, the rate has increased by 0.32% per year, while in high-income countries, it has decreased by 0.35% [10]. By gender, the global mortality rate in males has decreased from 0.55 to 0.32, and in females, it has decreased from 0.02 to 0.01 per 100,000 inhabitants. Specific studies conducted in some high-income countries align with these trends. For instance, in United States, the number of deaths from silicosis has seen an 85% reduction between 1968 and 2006, and in Italy, a 74% decrease was observed from 1990 and 2012 [11],[12]. In a middle-income country such as Brazil, the crude mortality rate exhibited a 2.77% annual increase between 1980 and 2006, followed by a 1.92% decrease until 2017 [13].

In the case of Spain, and following the definition provided by the European Commission, silicosis would be classified as a rare disease, affecting no more than 5 people in 10,000 inhabitants [14]. In addition, this disease remains highly relevant within the catalog of preventable respiratory pathologies. In 2019, silicosis in Spain accounted for 210 deaths and 2777 Disability Adjusted Life Years (DALY) [9]. Nationally, there exists a specific surveillance protocol dedicated to silicosis, outlining criteria for prevention and guidance for healthcare professionals [15]. Over the last 20 years, the incidence of silicosis has seen an increase in this country, particularly since the early 2000s, as indicated by various studies [16],[17]. However, no researchers have analyzed the spatial and temporal evolution of mortality related to this disease in Spain and to ascertain whether the observed rise in incidence has been related to increased mortality rates.

The mortality data, provided by the Spanish National Statistics Institute (NSI) (*Instituto Nacional de Estadística*), represent a high-quality and standardized source, enabling us to analyze trends over time. Furthermore, as the mortality data are individualized, they include a geographical variable, allowing for the examination of inequalities across the country. Geographic variables are essential for identifying health disparities within a territory and facilitating decision-making for political authorities to address inequities to the fullest extent. Hence, there is a plethora of scientific publications that incorporate support from geographic information system technologies in the field of health [18],[19]. Nevertheless, to date, neither epidemiological studies nor reports have addressed mortality due to silicosis at the national level in Spain.

Our aim in this study focuses on assessing the trend over time and the geographic distribution of mortality due to silicosis for the period 1999–2020.

## 2. Materials and Methods

Mortality data were obtained from the Spanish National Annual Death Registry, maintained by the NSI. Silicosis as an Underlying Cause of Death (UCD) was identified by J62.8 code from the International Classification of Diseases, 10th revision. Additionally, population data were obtained from the NSI, categorized by gender and five-year groups at both municipal and provincial levels for the study period.

The mean annual age at death was calculated for mortality and assessed using Joinpoint regression to identify trend change points for both sexes-men and women [20]. Annual age-adjusted mortality rates were calculated, with the European standard population as reference, for both genders, males, and for females (expressed per 1,000,000 inhabitants) [21]. The total deaths were divided into four age groups based on their number, in order to analyze the evolution of adjusted mortality rate over the period: Under 60, 60 to 69 years, 70 to 79 years, and over 80 years old. Joinpoint regression models were employed to evaluate annual trends for age-adjusted rates and age at death, identifying changing trends during the study period. For the temporal evolution by province, the annual age-adjusted mortality rates were calculated for each of the 52 provinces of Spain for both sexes, as well as the Annual Percentage Change (APC).

To spatially analyze the potential heterogeneous distribution of mortality at a more detailed level, we calculated the existence of clusters of deaths at the municipal level in two 11-years subperiods: 1999–2009 and 2010–2020. Poisson methodology was applied to calculate the relative risk, using circles of different sizes from the spatial centroid of each municipality [22]. Circular windows were computed ranging from a radius of 0 kilometers to an unspecified maximum distance for the centroid of each municipality. This is be detected by the algorithm when it finds that the number of observed cases within that circle exceeds the expected number of cases. Each cluster comprises a circular radius as well as a varying number of municipalities. This method analyzes through the number of cases and the population at risk in an area compared to its surroundings. The result for each cluster found is indicated by the value of the relative risk (RR) of the aggregated municipalities.

Statistical analyses were performed using R, Joinpoint and SatScan software, while QGIS was employed for cartographic representations.

## 3. Results

There were 2618 silicosis-related deaths in Spain from 1999 to 2020. Male mortality far exceeded that of females, with men accounting for 98.5% of deaths (2573 *vs*. 38 women). A decrease of 61.8% was observed from 178 deceases in 1999 to 68 in 2020.

The average age of death increased from 75.2 years in 1999 to 80.8 years in 2020. Join point analysis revealed a yearly increase of 0.66% (*p* < 0.001) from 1999 to 2013, and it has remained stable thereafter. For males, the age at death shifted from 75.25 to 81.05 years, experiencing a significant annual increase of 0.66% from 1999 to 2013 (*p* < 0.001).

**Figure 1.**
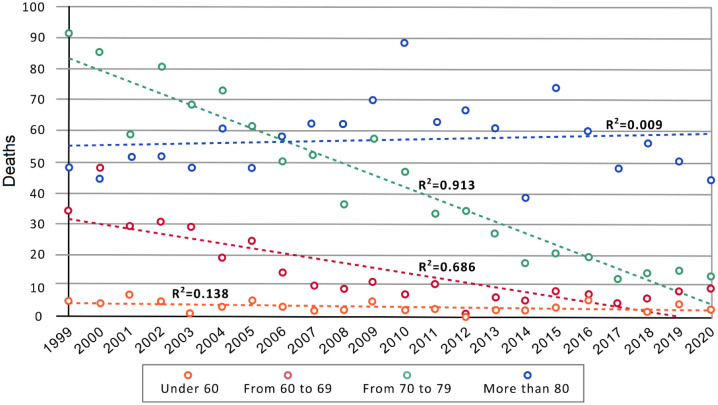
Number of deaths with silicosis as Underlying Cause of Death per age groups. Line trend is added.

Figure 1 illustrates the annual distribution of cases by age group. Deaths under 60 (*n* = 69) and those over 80 years of age (*n* = 1257) have shown a stable trend, with minimal variations. However, in the age groups of 60 to 69 (*n* = 329) and particularly 70 to 79 years (*n* = 963), there has been a decrease in the recorded silicosis deaths over the period.

**Figure 2.**
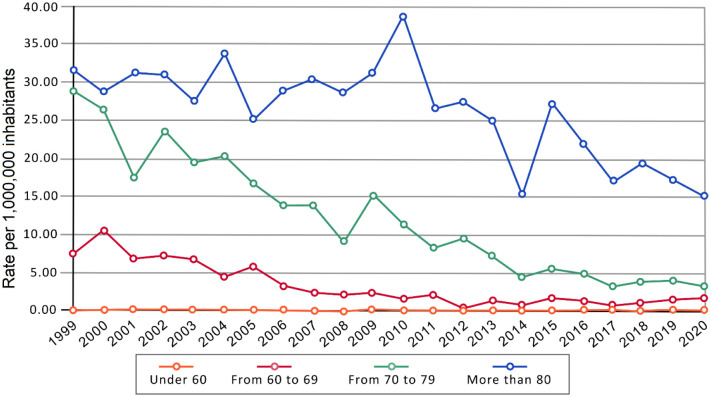
Annual adjusted mortality rates per 1,000,000 inhabitants in both sexes by age group.

For Spain, the adjusted mortality rate due to silicosis has decreased from 3.00 in 1999 to 0.65 per 1,000,000 inhabitants in 2020. In men, it has decreased from 7.00 per 1,000,000 to 1.50, while it has remained stable in women between 0.00 and 0.05. The adjusted mortality rate has decreased in both sexes and in men by 7.30% annually (*p* < 0.001), and in women, there was a decrease of 0.45% (*p* = 0.98). Figure 2 shows this trend by age group at death. Mortality rates have decreased significantly, except in those under 60 years (*p* = 0.202). In the age group between 60 and 69 years, the annual age-adjusted mortality rates has dropped from 10.65 in 2000 to 0.20 per 1,000,000 in 2012. Throughout the period, there has been a significant annual decrease of 10.73% (*p* < 0.001) in both sexes and 11.06% decline in men (*p* < 0.001). In women, it increased by 3.79% (*p* = 0.75). In 70–79 age group, the annual age-adjusted mortality rates has gone from 28.82 to 3.19 per 1,000,000, revealing an annual decrease of 10.13% (*p* < 0.001). In males, the rate dropped from 67.70 to 5.90 per 1,000,000 in the study period (-10.64%, *p* < 0.001). The rate increased in women by 5.03% (*p* = 0.74). In those over 79 years of age, in both sexes, the annual age-adjusted mortality rates has gone from 31.56 to 15.04 per 1,000,000, decreasing by -2.99% in the study period (*p* < 0.001). This trend was observed in males (dropping from 95.54 to 42.13 per 1,000,000; -3.43% per year, *p* < 0.001) and in women (-2.59%, *p* = 0.87).

In the study period, 17 out of the 52 Spanish provinces had significant changes in mortality due to silicosis (Table 1). A decrease was observed in 16 provinces, four with APC exceeding 25% (Huelva, Valladolid, Girona and Almería). Only the province of Pontevedra have an increase in mortality due to silicosis in this period.

Of the total number of cases recorded, 1616 (61.9%) corresponded to 1999–2009, and 994 (38.1%) to 2010–2020. For both sexes combined, in the first period, 225 municipalities aggregated in 11 clusters distributed across the territory were found to have a significantly higher mortality than expected. The number of municipalities increased to 347, distributed in the same number of clusters for the second period. The numerical results of the detected clusters in the two periods are shown in Table 2.

**Table 1.** Statistically significant Annual Percentage Change (APC) in age-adjusted mortality rate by province for the period 1999–2020.

**Province**	**APC (95% Confidence Interval)**	***p*-value**
Huelva	-30.3 (-42.1 to -16.1)	0.001
Valladolid	-29.9 (-42.9 to -13.7)	0.002
Girona	-27.8 (-42.5 to -9.2)	0.008
Almería	-25.0 (-42.2 to -2.5)	0.033
Salamanca	-24.7 (-36.3 to -11.0)	0.002
Zamora	-23.7 (-41.2 to -1.0)	0.043
Segovia	-22.7 (-39.1 to -1.9)	0.035
Jaén	-18.9 (-31.6 to -3.8)	0.019
Alicante	-16.3 (-25.5 to -5.8)	0.005
Madrid	-15.4 (-23.6 to -6.3)	0.003
Murcia	-14.4 (-24.1 to -3.5)	0.014
León	-9.5 (-11.3 to -7.6)	<0.001
Asturias	-9.0 (-10.6 to -7.3)	<0.001
Barcelona	-8.5 (-11.2 to -5.7)	<0.001
Vizcaya	-7.8 (-11.4 to -4.0)	<0.001
Palencia	-7.1 (-11.1 to -3.0)	0.002
Pontevedra	+6.4 (2.7 to 10.2)	0.001

**Table 2.** Silicosis mortality clusters by subperiod in Spain: a) Subperiod 1 (1999–2009); b) Subperiod (2010–2020).

**a) 1999–2009**							
**Cluster**	**Radius (km)**	**Municipalities affected**	**Provinces included**	***p*-value**	**Observed cases**	**Expected cases**	**RR (IC 95%)**
1	91.5	79	6	<0.001	450	37.8	16.1
2	121.2	29	6	<0.001	66	11.9	5.8
3	48.0	7	1	<0.001	34	6.1	5.7
4	61.0	11	2	<0.001	12	0.5	25
5	32.2	8	2	<0.001	18	1.8	10.3
6	24.2	10	1	<0.001	97	45.5	2.2
7	0	1	1	<0.001	11	1.1	10.0
8	98.5	19	5	<0.001	55	24.3	2.3
9	71.6	29	5	0.002	41	17.2	2.4
10	36.8	21	2	0.009	32	12.8	2.5
11	38.1	11	2	0.014	14	3.2	4.4

**b) 2010–2020**							
**Cluster**	**Radius (km)**	**Municipalities affected**	**Provinces included**	***p*-value**	**Observed cases**	**Expected cases**	**RR (IC 95%)**

1	328.0	151	9	<0.001	480	120.0	6.8
2	144.7	87	8	<0.001	343	56.0	8.8
3	103.9	54	4	<0.001	119	42.7	3.0
4	115.9	15	4	<0.001	27	3.0	9.2
5	37.0	5	3	<0.001	8	8.5	18.0
6	24.6	8	1	<0.001	11	1.2	9.5
7	11.2	6	1	<0.001	13	2.0	6.8
8	91.1	9	3	<0.001	10	1.1	9.6
9	64.7	5	2	0.006	5	0.3	19.7
10	45.9	6	2	0.012	9	1.3	7.0
11	0	1	1	0.038	2	0.0	156.7

**Figure 3.**
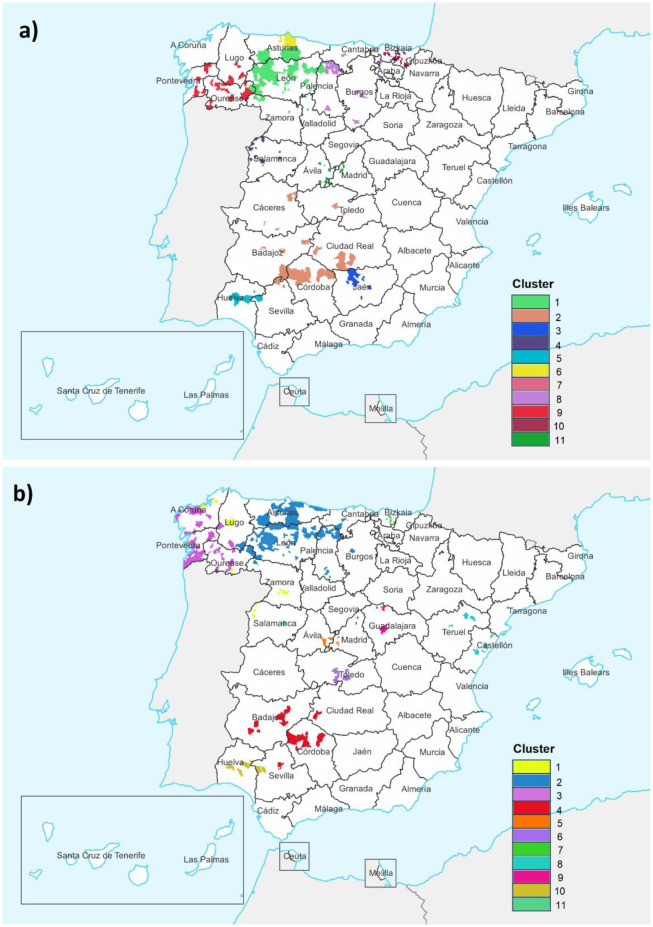
Mortality clusters detected by municipality: a) Subperiod 1999–2009; b) Subperiod 2010–2020. Names of provinces are shown to enable identification.

As can be seen in the Figure 3, mortality clusters detected in the first subperiod were located throughout the western fringe of Spain, both in the north and in the south. This pattern was found in the second subperiod but with variations in some provinces.

In the north of Spain, when comparing both subperiods, a cluster located in the provinces of Pontevedra and Ourense (*RR* = 2.42, *p* = 0.002) shifted westward to the provinces of Pontevedra and A Coruña (*RR* = 3.03, *p* < 0.001). Different groups of clusters are concentrated in the northern sector, covering the provinces of Asturias, León and northern Palencia, comprising a high number of municipalities. Another cluster in Bizkaia and Gipuzkoa (*RR* = 2.54, *p* = 0.009) was limited only to the first-mentioned province in the second period (*RR* = 6.77, *p* < 0.001).

In central Spain, in addition to the clusters in the subperiod 1999–2009, new areas have appeared in the provinces of Guadalajara and Toledo. In eastern Spain, a small cluster of cases has emerged in the provinces of Teruel and Castellón (*RR* = 9.61, *p* < 0.001).

In the south of Spain, one cluster remains in the north of the province of Cordoba and its surroundings, as well as in the province of Huelva. On the other hand, two small clusters detected in the first subperiod in the provinces of Jaén (*RR* = 5.65, *p* < 0.001) and Murcia (*RR* = 9.96, *p* < 0.001) disappeared in the second one.

Regarding sex, since men account for practically all the deaths, the cluster distribution is practically the same as that observed for both sexes, as is the spatial variation between subperiods. In women, only one cluster was found in the first subperiod corresponding to the municipality of Vigo in the province of Pontevedra in northwestern Spain (*RR* = 40, *p* = 0.001), while none were detected in the second subperiod.

## 4. Discussion

We demonstrate the decrease in silicosis mortality during the period 1999–2020 and its geographical distribution in Spain, comparing two 11-year subperiods. In this manner, an epidemiological study on silicosis has been carried out, proving highly valuable population-based data for complementing other studies already conducted on prevalence or regional analyses in Spain.

Spain, like other developed countries, has witnessed a significant decrease in silicosis mortality. Comparing the period 1999–2009 and 2010–2020, the number of deaths experienced a significant decline. Similar studies conducted in Italy, the United States, and Brazil in recent decades have reported a parallel trend [11]–[13]. The reduction in mortality is largely attributed to heightened awareness of this occupational disease and the implementation of preventive measures. In Spain, the National Institute of Silicosis [*Instituto Nacional de Silicosis*] (NIS), established in 1970, plays a crucial role in monitoring new cases and occupational factors associated with this disease [23]. The Ministry of Health initiated two surveillance protocols for silicosis in 2001, with an update in 2020 [15],[24]. This underscores the prioritization of occupational pathology in public health policies in Spain. The national protocol for primary prevention advocates maintaining respirable dust levels legislatively defined limits: Two simultaneous measurements of 3 mg/m^3^ of dust (respirable fraction) and 0.05 of respirable crystalline silica dust per day [25]. Secondary and tertiary prevention guidelines are also in place once silicosis is diagnosed.

Contrary to these mortality data indicating a decrease silicosis-related deaths, statistics on new patients diagnosed by the NIS remained stable in the 2010–2020 subperiod (-0.20% annually, *p* = 0.91). Annual reports revealed a minimum of 115 new cases in 2007, peaking at 270 in 2018 [26]. This stable trend in new cases, coupled with a simultaneous decline in mortality, leads us to hypothesize that the survival of patients with silicosis may have been increasing for many decades. In a survival analysis conducted in a Chinese city, patients with silicosis exhibited higher survival rates if they were non-smokers and younger than 30 years old [27].

In the case of the mortality rate by province, there has been a significant decrease in 16 out of 52. Pontevedra stands out as the province that has significantly increased its rate. As shown in a recent study, the number of new reports of silicosis cases has increased between 2009 and 2019 in the Galicia region (where Pontevedra is), particularly in the manufacturing, machining, and installation of quartz conglomerates [16]. It is possible that this progressive increase in silicosis among workers is being reflected in mortality rates in this province.

It is well known that silicosis is a disease that traditionally affects miners. It is a chronic and long-lasting condition, which means that mortality rates remain high in traditional mining areas despite a significant reduction in activity over the last 20 years, nearly disappearing. The number of mine workers has decreased from over 120,000 in 1985 to less than 20,000 in 2002, and by 2018, this figure has not exceeded 2000 workers [28],[29]. Consequently, in these former coal mining areas (Asturias, northern Leon and Palencia) mortality is high. However, other clusters are emerging in places unrelated to traditional mining, linked to more contemporary activities associated with silica exposure, such as the production of artificial stone countertops. According to the NIS, 70% of the newly detected cases were workers or former workers in the coal sector in 2009, but for the year 2000, this percentage dropped to 24%. The main occupation of the affected workers was the slate sector (49.1%) and, to a lesser extent, granite and marble (8.5% and 7.9%, respectively) [26]. In a district in the province of Almería (southwest of Spain) specializing in mining and marble industry, it was found that assemblers of home kitchen countertops were the most affected by silicosis due to exposure to silica dust and the lack of respiratory protection measures [30]. No cluster of mortality cases has been identified in this area, but it could occur in the future.

In a study on occupational diseases reported in Spain, which includes silicosis, an increase in RR is evident from 1999 to 2011 in Galicia region (where Pontevedra is) [31]. Conversely, RR has decreased in Asturias, as well as in Castile and León (which includes León, Salamanca, Palencia and Zamora among other) and Basque Country (including Vizcaya). This decrease aligns with the results showed in the present study. Notably, the report highlights consistently high RR in La Rioja across all observed periods, although this province has not shown a significant evolution in mortality in this study.

Some authors indicate that mining and slate manufacturing are among the occupations associated with the development of silicosis [32],[33]. In Spain, starting from 2016, workers in the slate industry accounted for more than 50% of new silicosis cases, whereas previously, coal mining was the predominant sector [26]. We identified some of the main clusters of silicosis mortality Galicia region (northwestern Spain) and the western part of León. This area is characterized by a significant number of slate exploitations, reaching about 70% of the total in Spain in 2010 [34]. Within Galicia, in a district of the province of Ourense (Valdeorras) where slate mining and manufacturing are the major industries, a small cross-sectional descriptive study was conducted on patients with occupational silicosis. Of the total number of patients, more than 95% reported not using personal protective measures such as FFP3 masks [35].

Observing the distribution of mortality rates across the provinces of southern Spain, a noteworthy decline has been observed in the province of Jaén, amounting to a significant annual decrease of 19% during the study period. Furthermore, a municipal cluster identified in the initial subperiod is no longer extant in the later subperiod. This reduction is likely attributable to the cessation of mining activities within this province over the course of the 20th century [36]. In contrast, in other Andalusian provinces, such as Huelva, have also experienced a decline in mortality rates, exhibiting a notable reduction of 30.3% per annum. However, it is noteworthy that municipal clusters of deaths persists in these areas, despite a reduction in mining activities. In these instances, mining has been curtailed but remains extant.

The results of our study aim to raise awareness about the necessary actions to be carried out for the public health community. Public health interventions focused on reducing the burden of disease and improving quality of life should consider three main aspects. First, health protection measures at workplaces or the community to maintain silica dust exposure under the threshold level set by the national legislation. Second, the implementation of health surveillance protocols at high-risk workplaces to identify affected individuals at early stages, slow the disease progression, and decrease the number of complications as cancer or arthritis. In addition, such programme could detect individuals with associated factors that would benefit from other preventive strategies as smoking cessation programmes or immunization schemes [15]. Third, population-based research on environmental and occupational exposures is a key strategy to identify vulnerability factors associated with silicosis (spatial and temporal trends, sociodemographic conditions, etc.) so that public health policies could be adapted to address those elements.

As limitations of this study, the mortality registry indicates the place of residence at the time of death but does not provide information regarding whether the person lived elsewhere previously. This should be considered a constraint when establishing a direct cause-effect relationship between the place of residence during their working life and subsequent changes. One the other hand, one of the strengths of this study is that the raw data with which we have worked come from a national population-based registry, which has remained stable over time and employs a standardized methodology. As a limitation, it should be mentioned that there could be an underestimation of cases of deaths due to silicosis in its registration as UCD. Instead, it might be registered as other diseases that are commonly associated, such as chronic obstructive pulmonary disease, chronic bronchitis, lung cancer, rheumatoid arthritis, or kidney failure. In the case of lung cancer, the International Agency for Research on Cancer, as well as Spanish legislation, indicates that occupational exposure to silica dust at the workplace is a carcinogenic agent [24],[37]. Although the survival of individuals with silicosis has increased, it is likely that if they have associated diseases, the quality of life of the patients could be deteriorating as these conditions are highly disabling.

## 5. Conclusions

In conclusion, the results are very positive regarding the decrease in mortality in Spain due to silicosis in the last 22 years. However, the importance of personal hygiene measures, individual protection, as well as risk minimization actions by companies, such as ventilation of enclosed spaces, or moistening materials, among others is remarkable. The decrease in mortality is likely attributed to the increased survival rate of the affected individuals. In the future, it will be necessary to monitor the potential impact on mortality of the reported increase in the incidence, as observed in Spain and in other studies.

## Use of AI tools declaration

The authors declare they have not used Artificial Intelligence (AI) tools in the creation of this article.
